# The influence of a vegan diet on body composition, performance and the menstrual cycle in young, recreationally trained women– a 12-week controlled trial

**DOI:** 10.1080/15502783.2024.2413961

**Published:** 2024-10-07

**Authors:** Eduard Isenmann, Isabella Trojak, Alessio Lesch, Jan Schalla, Tim Havers, Patrick Diel, Stephan Geisler

**Affiliations:** a IST University of Applied Sciences, Department of Fitness and Health, Dusseldorf, Germany; b German Sport University Cologne, Institute for Cardiovascular Research and Sports Medicine, Department of Molecular and Cellular Sports Medicine, Cologne, Germany

**Keywords:** Vegan diet, menstrual cycle, plant-based diet, physical performance, body composition, recreationally trained women

## Abstract

**Background:**

An increasing number of people, including recreational trained individuals, choose not to consume animal products and follow a vegan diet. Young women in particular are switching to a vegan diet. Studies have shown no difference in performance and muscle adaptations between a balanced vegan and an omnivorous diet. However, there are hardly any studies on the transition phase from an omnivorous to a vegan diet and the potential difficulties. Therefore, this study aimed to investigate the influence of a vegan dietary transition and its effects on body composition, physical performance, and menstrual cycle in young, recreationally trained women.

**Methods:**

Ten young healthy women (23.8 ± 2.0 years, 173.0 ± 5.8 cm) were recruited to participate in this 12-week controlled study (4-week omnivorous phase, 8-week vegan intervention). At the beginning and before the vegan phase, all participants were informed about a balanced diet for fitness-oriented individuals and a vegan lifestyle. They were supervised by a sports dietitian for the entire 12 weeks. Explicit instructions and regular checks on macronutrient distribution were not carried out but had to be implemented independently. The diet was documented using FDDB Extender. The training habits were not explicitly specified, but should not be changed over the entire period. At baseline (T0) and 4-week intervals (T1, T2, T3), body composition (body weight, skeletal muscle mass, fat mass) and performance (squat, countermovement jump) were tested. In addition, the menstrual cycle was examined every two days using saliva samples and a cycle diary.

**Results:**

Between treatments, there was a significant decrease in absolute (T0: 94.44 ± 20.37 kcal; T3: 71.67 ± 27.64 kcal; *p* < 0.001) and in relative protein intake (T0: 1.39 ± 0.28 g/kg BW; T3: 1.06 ± 0.37 g/kg BW; *p* < 0.05). In carbohydrate consumption, a significant increase was observed (T0: 240.11 ± 53.15 kcal; T3: 266.89 ± 49.01 kcal; *p* < 0.001). During the vegan phase, a significant decrease in body weight (T0: 68.19 ± 6.47 kg, T3: 67.73 ± 6.07 kg; *p* < 0.001) and skeletal muscle mass (T0: 29.40 ± 2.23 kg; T3: 28.74 ± 2.55 kg; *p* < 0.001) was observed. No changes were noted in squat performance. The countermovement jump showed a significant decrease in the vegan phase (T0: 26.08 ± 3.44 cm; T3: 23,62 ± 1,00 *p* < 0.05), but also a significant time effect starting in the omnivorous phase (*p* < 0.001). No effects were found on hormone concentrations of individual menstrual cycles.

**Conclusion:**

The dietary change resulted in a shift in overall macronutrient distribution. Relative protein intake was significantly lower during the vegan phase than during the omnivore phase. This was also observed in a slight decrease in skeletal muscle mass. No clear effects on performance and menstrual cycle were observed during the first eight weeks. The results suggest that despite the knowledge of a balanced diet and in particular the recommendations for a vegan diet, the implementation of a vegan diet in everyday life could be associated with a number of difficulties for recreationally trained women. However, it should be noted that the vegan phase was only conducted for eight weeks and no statement can be made about the long-term effects or on well-trained female athletes.

## 1. Introduction

A balanced diet is often associated with a healthy lifestyle and overall well-being. For some years, the consumption of animal foods such as meat, fish and dairy products has been discussed extensively. A dietary strategy which excludes all kinds of animal products is the vegan diet [1]. It promises various effects on the cardiovascular system, for example, the cardio-metabolic risk profile [2] and blood creatine phosphate levels have been positively affected by a vegan diet [3]. Furthermore, it could be demonstrated that due to the anti-inflammatory effect of a vegan diet, there are preventive effects on the development of obesity, hypertension, diabetes, and cardiovascular mortality [4–6]. Other reasons for dietary change include ethical, ecological, or social issues [7].

However, there are also some concerns with a vegan diet. One aspect is the critical need for an adequate intake of vitamin B12 [8], which is essential for the breakdown of fats, cell metabolism, and mental development [8]. A deficiency can lead to an accumulation of the amino acid homocysteine and thus to possible risks of cardiovascular diseases [8]. Furthermore, a vegan diet is often associated with a low energy density [9]. This can lead to various energy availability issues, especially for fitness-oriented individuals and athletes [9,10]. In addition, initial observations show that vegans have a lower protein intake than omnivores in the general population [7].

The energy and protein requirements of recreational, fitness-oriented, and competitive athletes are higher than those of the general population, depending on the duration of training [11–14]. In addition, there are further recommendations for physically active individuals and athletes who follow a vegan lifestyle to meet their daily protein requirements [10]. To maintain a balanced amino acid profile, it is recommended to combine cereals and legumes. Foods such as beans and pulses are rich sources of lysine, and leucine can be obtained from soya beans and lentils [10].

Currently, there are only a few studies on the effects of a vegan diet on performance and body composition. Initial studies have found no differences in protein biosynthesis or muscle growth when energy expenditure and protein intake are equalized between omnivorous and vegan diets in combination with systemic strength training [15,16]. In both studies, a high protein intake of at least 1.6 g/kg BW was specified for the vegan and omnivorous groups over the entire intervention period. Hevia-Larraín et al. found a significant increase in skeletal muscle mass (SMM) in untrained individuals with two strength training sessions per week after 12 weeks, with no difference between the dietary strategies [15]. Monteyne et al. found similar results in trained individuals with 5 training sessions per week for 10 weeks [16]. Furthermore, current reviews show no differences in performance and activation of signaling cascades between an omnivorous and a vegan diet [17–19]. However, the majority of studies focus exclusively on matched energy intake or macronutrient distribution, or compare the performance of athletes who have already implemented their nutritional habits in their everyday lives. Only one pilot study aimed to identify potential difficulties in switching to a vegan phase [20]. The authors found that despite advice from a nutrition coach, without direct guidance on energy intake or macronutrient distribution, protein intake decreased significantly during the vegan phase in recreationally trained individuals [20]. However, no significant changes in strength capacity were observed in the first eight weeks in this population [20]. The observations of a lower protein intake are in line with previous studies on the general population [7]. It seems that despite the independent documentation of the diet and the requirement to adhere to the total daily calorie needs and macronutrient distribution, the participants were not able to implement this in their daily lives. It appears that further difficulties can arise when changing eating habits to a vegan diet. It is not yet clear what effect the transition to a vegan diet has on higher performance levels.

In terms of the population most likely to adopt to a vegan lifestyle, recent research has identified young women [21,22]. In general, the database on women in the sports medicine context is extremely limited and scarce regarding the influence of a vegan diet on health, body composition, and performance [23].

Based on the limited data available, this is the first study to focus specifically on recreationally trained women and the effects of switching from an omnivorous to a vegan diet without specific macronutrient intake requirements on body composition, lower body performance, and the menstrual cycle.

## 2. Materials and methods

### 2.1. Participants

Young, healthy female participants aged 21–30 years with at least two months of consecutive strength training and a total of at least two years of strength training experience were recruited from the area around Münster, Germany. Other physical activities, such as endurance training, attending fitness classes or team sports, were also permitted and documented over the period. Participants’ general performance level was categorized as recreationally active to trained [24]. In terms of strength training ability, the participants were at least at an intermediate level [25]. Eligibility criteria included performing the squat at least once a week during training, having a regular menstrual cycle for at least three months prior to the study, not using contraceptives, and following an omnivorous diet. Individuals who were already following a vegan or vegetarian diet were not allowed to participate in the trial. Based on previous studies [20] and the high number of measurement points for nutrition and the menstrual cycle, ten female participants initially took part in the study.

### 2.2. Study design

**Figure 1.**
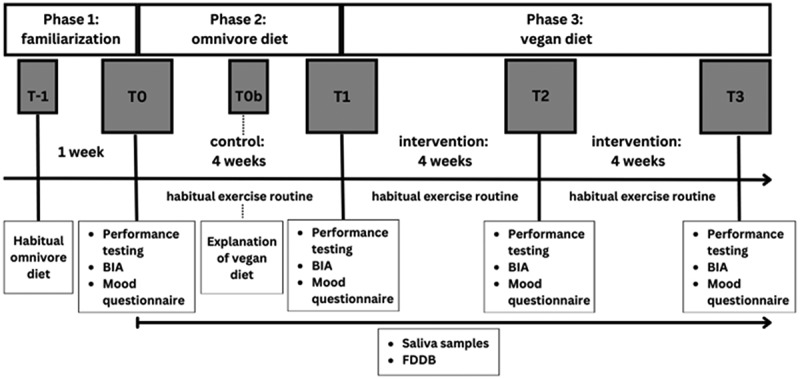
Schematic representation of the study design. BIA = bioelectrical impedance analysis. The performance tests consisted of the countermovement jump and the 1RM back squat. Saliva samples were collected every second day.

This study was approved by the local ethics committee of the German Sports University Cologne, Germany (125/2021) and entered into the German register for clinical trials (DRKS00031633). The study design was divided into three phases (familiarization, omnivore and vegan phase), four measurement points (T0, T1, T2, and T3), and lasted 12 weeks. Prior to the start, all participants were educated about a balanced diet for recreationally trained and fitness-oriented individuals (*T*-1). The nutritional recommendations are based on position papers of the International Society of Sports Nutrition (ISSN) [11,14]. This was followed by a one-week familiarization phase (Phase 1), in which participants were familiarized with the documentation of their diet in the Food Diary Food Database FDDB (version 2.3.17, Food Database GmbH 28,217 Bremen). After a 4-week omnivorous phase (Phase 2), all participants were informed about a vegan diet according to the current recommendations for physically active individuals and athletes (T0b) [10]. This was followed by an eight-week vegan phase (Phase 3). Saliva samples and menstrual cycle monitoring were also carried out during the entire period. Subsequently, power and strength measurements, anthropometric data, and questionnaires on mood, energy levels, and cycle-specific symptoms [26,] were collected at time points T0, T1, T2 and T3. The full study design is illustrated in Figure 1. The participants maintained their usual weekly training regimens. However, participants were encouraged to document the type of sport, intensity, and frequency of their exercise. Exercise habits were not influenced and were maintained throughout the study.

### 2.3. Dietary strategies and documentation

#### 2.3.1. Diet

At the start of the study, all participants followed an omnivorous diet. During the omnivorous phases (Phases 1 and 2), the subjects were allowed to consume all foods of animal and plant origin. Participants were advised to follow the general ISSN’s recommendations for fitness-oriented individuals and athletes [22] but no clear guidelines regarding calorie intake, macronutrient distribution, or food quality were given. The ISSN’s current general percentage recommendation for macronutrient distribution are 45–55% carbohydrate, 15–20% protein and 15–25% fat [11].

An explanation of a vegan diet (T0b) and the current recommendations for physically active individuals and athletes was followed by an eight-week vegan phase (Phase 3) [10]. All meat and dairy products, as well as any other animal-based products, such as eggs or honey, were prohibited during the entire period. Furthermore, foods whose production involves animal products were eliminated from the diet. As in Phase 2, no clear guidelines were set for energy intake and macronutrient distribution. All participants were supported by a nutrition coach throughout the entire study period. The nutrition coach was in contact with the participants several times a week to answer potential questions and monitored the participants’ nutrition logs. The nutrition coach only actively intervened if there were missing entries in the diet or if there was a significant calorie deficit. However, no information was provided on the difference between the omnivorous phase and the vegan phase. The participants were independently responsible for maintenance of energy requirements and macronutrient distribution between Phase 2 and Phase 3 and for implementing this in their everyday lives. The protocol used was similar to the one used in a previously published study [20].

#### 2.3.2. Nutrition documentation

During the entire 12-week period, the diet was monitored using the FDDB Extender. All foods and beverages consumed during the day had to be documented. The FDDB Extender is an established method for accurately recording macronutrient distribution [27]. The FDDB Extender has therefore been used in previous nutritional interventions [20,28].

### 2.4. Measurements

#### 2.4.1. Anthropometric parameters

At each time point (T0-T3), body weight (BW), skeletal muscle mass (SMM), and fat mass (FM) were determined using bioelectrical impedance analysis (BIA; InBody 570, InBody Europe B.V., Germany 65,760 Eschborn). Using an 8-electrode approach, a total of 15 impedance measurements were obtained. Three frequencies each for the upper and lower extremities and the trunk. To maintain consistency in the measurements, the participants refrained from engaging in any physical activity two days beforehand. They also avoided alcohol for one day prior and refrained from consuming any dehydrating drinks two hours beforehand. Additionally, they made sure to have the same breakfast meal and wear similar clothing. Each measurement was taken between 9:00 and 12:00 a.m.

#### 2.4.2. Documentation of menstrual cycle

To verify the menstrual cycle, a cycle diary (documentation of cycle length, length of period, symptoms during the period) and saliva samples were collected. Saliva analyses are valid for identifying irregular hormone fluctuations, but not for identifying absolute concentrations [29]. In this study, the focus was on the identification of abnormal menstrual cycle rhythms. Saliva samples were collected from each participant every two days from the beginning of the experiment. The saliva sample was collected in the morning directly after getting up. The ingestion of liquids (water, coffee, tea, etc.) as well as brushing teeth was prohibited before saliva collection. Specific ELISA kits for estradiol (E2) and progesterone (P) concentrations were used (RE52281 and RE62141, respectively). Each subject was provided with a sufficient number of micro reaction vessels as well as small straws and stickers for labeling. The containers were filled at least halfway with clear saliva. Each sample was labeled with the subject code, date, sample number, and time, and then mandatorily stored in the freezer (−20°C). The samples were then handed to the investigator at the end of the study period.

#### 2.4.3. Jumping ability – countermovement jump

Before the measurement, a 10-minute warm-up and one submaximal test jump were performed. Subsequently, the participants performed three countermovement jumps (CMJ). A rest period of 60 seconds was provided between each trial for recovery. CMJ measurements were performed using the Optojump System (Microgate, Bolzano, Italy), as previously described [30]. The mean of all three trials was used in the analysis.

#### 2.4.4. One-repetition maximum in barbell back squat

To test the maximum strength of the participants, a one-repetition maximum (1RM) test was performed on the barbell back squat. The test was always performed at the same gym using the same equipment, at the same time, and on the same day of the week. The test protocol was executed according to the NSCA guidelines [31]. After a 5-minute individual warm-up, the first specific warm-up set was performed with a 20 kg barbell and 15 repetitions. This was followed by 10 repetitions at 50%, 4–8 repetitions at 70%, and 2–4 repetitions at 80% of 1RM. A 1RM attempt was considered valid if the weight was independently lifted from the deep back squat position (defined as squatting below 90° of knee flexion) back to standing. The 1RM attempts were staggered in 5 kg increments. If an attempt failed, weight was reduced by 2.5 kg. If the weight could then be lifted, another attempt was made using the last failed weight. In case of another failure, the measurement was completed. A break time of 3 min was provided between the warm-up sets and a 4-minute rest period was provided between the 1RM attempts.

#### 2.4.5. Questionnaire on mood, energy levels, and cycle-specific symptoms

The Matthey Generic Mood Questionnaire was used to assess well-being and energy level, and the questionnaire by Chesney et al. was used to survey symptoms during the menstrual cycle [26]. The items from both questionnaires were combined into one document.

### 2.5. Statistical analysis

The data was analyzed using the statistical software R (version 4.1.1) [32]. Linear mixed-effects (LME) models were used to investigate the effects of treatment and time on body composition (BW, SMM, FM), physical performance (1RM, CMJ) as well as the hormone parameters (E2, P). To all models, the cycle phase was added as a covariate of no interest. The effect of treatment and time on macronutrient distribution (carbohydrates, fats, proteins) as well as total energy uptake was also analyzed. Every parameter of interest was tested once for treatment differences (omnivore vs. vegan) and for differences over Time (T0-T3).

The lme4 package [33] was used to fit the LME model using the lmer function. Normal distribution was visually tested using a Q-Q plot. Hormone parameters (E2, P) were transformed into their natural logarithm to achieve normality.

A backward hierarchical modeling approach was used for the random effects. The fixed effects were not reduced, due to the interest in treatment and time alterations and still controlling for cycle phase. All presented models contain these two fixed effects. An exception are the models for the macronutrients were only treatment or time are included as fixed effects. Model comparisons were conducted via an ANOVA and the Akaike information criterion (AIC). If the AIC did not show a difference of at least 1 Unit, the Model with the lower AIC was accepted. The random effects allowed the intercept and the slope to vary for each individual and cycle phase based on the diet or time point. An initial significantly difference for the coefficients of the final model was set *p* < 0.05.

Effect sizes (d) were calculated using a modified version of Cohen’s d for mixed effect models [34]. Effect sizes are classified as trivial (d < 0.2), small (0.2≤d < 0.5), medium (0.5≤d < 0.8) or large (0.8>d) [35].

## 3. Results

A total of 9 out of 10 women (23.8 ± 2.0 years, 173.0 ± 5.8 cm) successfully completed the study. One participant had to be excluded two weeks after the start of the vegan phase because the dietary change triggered previous disordered eating.

### 3.1. Nutrition

**Figure 2.**
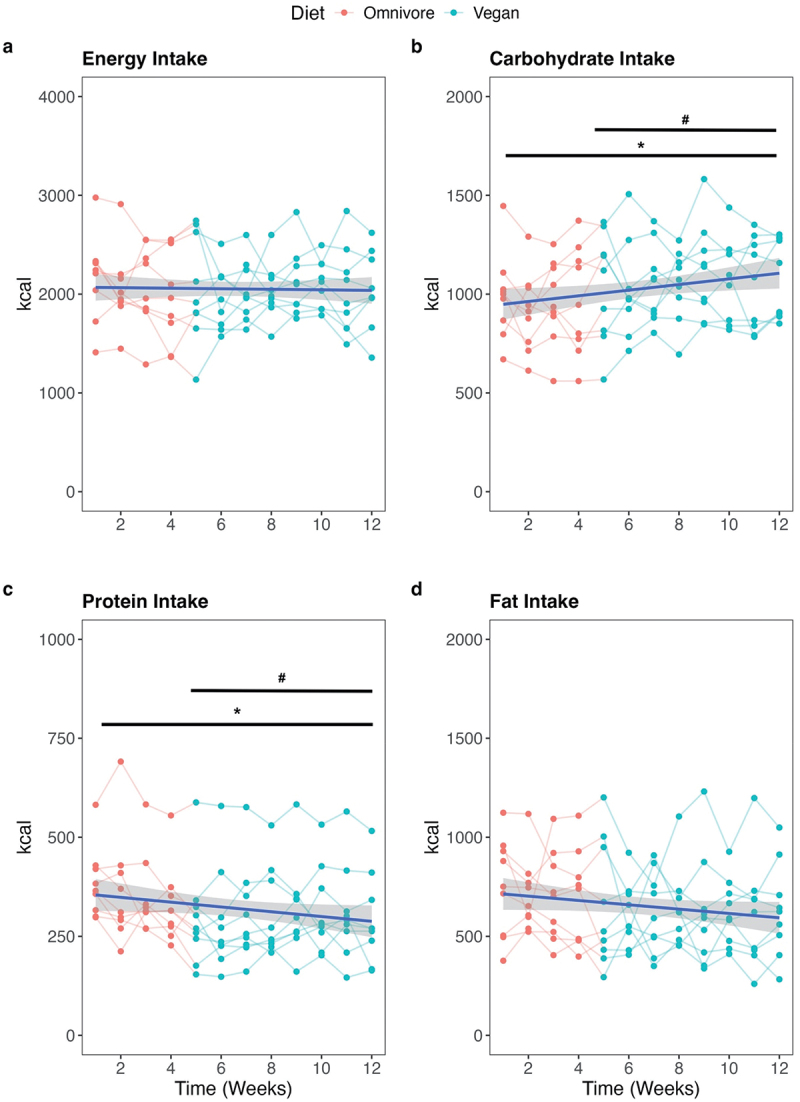
Means of absolute value (kcal) for energy intake (a), carbohydrate (b), protein (c), and fat (d). Blue lines show the locally weighted mean values (loess) with standard deviations and the colored lines the individual courses. Red highlights show the omnivorous phase and the blue highlights show the vegan phase. Significant time differences were indicated with * and treatment differences with #.

No significant change in self-documented energy intake was identified by the dietary change from an omnivore to a vegan diet (Figure 2a). However, a change in macronutrient distribution could be identified. For carbohydrates, a significant time (*p* < 0.01) and treatment effect was observed for total intake (*p* < 0.01, d = 0.46) (Figure 2b) and for relative intake (Table 1). In addition, a time and treatment difference in protein intake was observed. Significantly less protein is consumed during the vegan phase (time: *p* < 0.01; treatment: *p* < 0.01, d = 0.48) (Figure 2c). This is supported by the relative values (Table 1). No significant reduction was found in the absolute and relative fat intake over time and due to treatment. All average values of energy intake and macronutrients for each week can be found in Table 2.

**Table 1.** Relative values of macronutrient intake. *Mean value with standard deviation*. Significant differences were set *p* < 0.05 and marked for time with * and for treatment with #.

	Omnivore phase (4 weeks)		Vegan phase (8 weeks)		Time	Treatment	Effect size d
	T0	T1	T2	T3	T0–T3	T0–T1 vs T2–T3	Treatment
Protein (g/kg BW)	1.39 ± 0.28	1.18 ± 0.31	1.07 ± 0.37	1.06 ± 0.37	<.05*	<.05#	.66
Carbohydrate (g/kg BW)	3.57 ± 0.97	3.52 ± 1.06	3.88 ± 0.74	4.18 ± 0.85	<.01*	<.01#	.43
Fat (g/kg BW)	1.19 ± 0.42	1.09 ± 0.33	1.04 ± 0.24	1.08 ± 0.32	n.s	n.s	—

**Table 2.** Average energy intake and macronutrient distribution in each week. Mean value with standard deviation.

	Energy Intake (kcal)	Carbohydrates (g)	Fat (g)	Protein (g)
Week 1	2174.89 ± 436.10	240.11 ± 53.15	80.56 ± 26.85	94.44 ± 20.37
Week 2	2046.22 ± 388.64	221.22 ± 50.42	76.00 ± 19.91	89.78 ± 33.65
Week 3	2058.89 ± 416.14	234.44 ± 52.15	75.11 ± 24.39	85.56 ± 24.11
Week 4	1988.78 ± 475.77	236.00 ± 67.08	74.11 ± 25.78	79.78 ± 23.65
Week 5	2059.11 ± 545.39	252.67 ± 66.92	71.22 ± 33.95	72.11 ± 30.56
Week 6	1967.11 ± 313.29	248.22 ± 59.04	65.44 ± 17.53	70.33 ± 32.66
Week 7	2030.33 ± 301.56	256.44 ± 47.57	66.67 ± 21.71	74.11 ± 30.20
Week 8	2031.11 ± 280.12	255.78 ± 43.54	68.33 ± 21.87	74.56 ± 27.21
Week 9	2117.44 ± 338.49	272.11 ± 58.25	66.78 ± 30.32	77.22 ± 28.60
Week 10	2101.11 ± 243.40	264.00 ± 51.27	66.67 ± 18.33	77.11 ± 26.44
Week 11	2019.67 ± 435.83	256.44 ± 56.36	65.22 ± 29.57	75.33 ± 29.72
Week 12	2042.22 ± 390.38	266.89 ± 49.01	67.89 ± 25.67	71.67 ± 27.64

### 3.2. Body composition

Significant time and treatment effects were observed for BW (time: *p* < 0.01, d = 0.06; treatment: *p* < 0.01, d = 0.12). In addition, significant time and treatment effects were observed for SMM (time: *p* < 0.01, d = 0.1; treatment: *p* < 0.01, d = 0.21). No significant differences could be identified for body fat. All data is shown in Table 3.

**Table 3.** Analyses of body composition. *Mean value with standard deviation*. Significant differences were set *p* < 0.05 and marked for time with * and for treatment with #.

	Omnivore phase (4 weeks)		Vegan phase (8 weeks)		Time	Treatment	Effect size d
	T0	T1	T2	T3	T0–T3	T0–T1 vs T2–T3	T0-T3
BW (kg)	68.19 ± 6.47	67.54 ± 6.24	66.90 ± 6.57	67.73 ± 6.07	<.01*	<.01#	.12
SMM (kg)	29.40 ± 2.23	29.20 ± 2.21	28.69 ± 2.29	28.74 ± 2.55	<.01*	<.01#	.21
FM (kg)	15.48 ± 3.15	15.24 ± 3.20	15.39 ± 3.26	16.00 ± 2.22	n.s	n.s	—

### 3.3. Performance

In Table 4 the descriptive data for training frequency and training type are shown.

**Table 4.** Descriptive data of training frequency and training type. TS = training sessions, TS = ranged between 60-120 min, exact training hours per week were not monitored).

	Omnivore phase (T0-T1)	Vegan phase (T1-T3)
Strength training (TS/week)	1.88 ± 0.21	2.27 ± 0.35
Endurance training (TS/week)	2.14 ± 0.41	2.52 ± 0.27
Other physical activity (TS/week)	1.34 ± 0.39	1.23 ± 0.21
Training frequency (TS/week)	3.67 ± 0.84	4.06 ± 0.26

The CMJ showed significant differences over time and treatment (time: *p* < 0.01, d = 0.26; treatment: *p* < 0.05, d = 0.53). No significant differences were observed for 1RM (Table 5).

**Table 5.** Analyses of the squat and jumping performance. Significant differences were set *p* < 0.05 and marked for time with * and for treatment with #.

	Omnivore phase (4 weeks)		Vegan phase (8 weeks)		Time	Treatment	Effect size d
	T0	T1	T2	T3	T0-T3	T0–T1 vs T2–T3	Treatment
1RM BS (kg)	56.94 ± 6.71	58.06 ± 6.47	58.13 ± 8.32	57.50 ± 5.77	n.s.	n.s	—
1RM BS (rel.) (kg/kg BW)	0.83 ± 0.15	0.85 ± 0.14	0.87 ± 0.17	0.86 ± 0.17	n.s	n.s	—
CMJ (cm)	26.08 ± 3.44	25.23 ± 3.22	24.43 ± 3.35	23.62 ± 1.00	<.01*	<.05#	.53

### 3.4. Hormones

**Figure 3.**
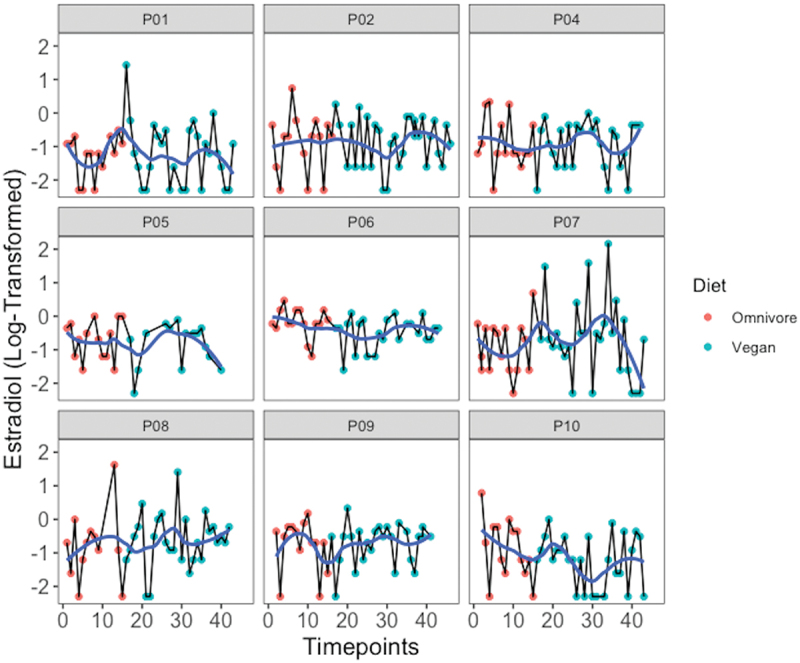
Estradiol values transformed to their natural logarithm over the 12 weeks for each individual. The blue line describes the locally weighted mean values (loess) to visualize time course.

**Figure 4.**
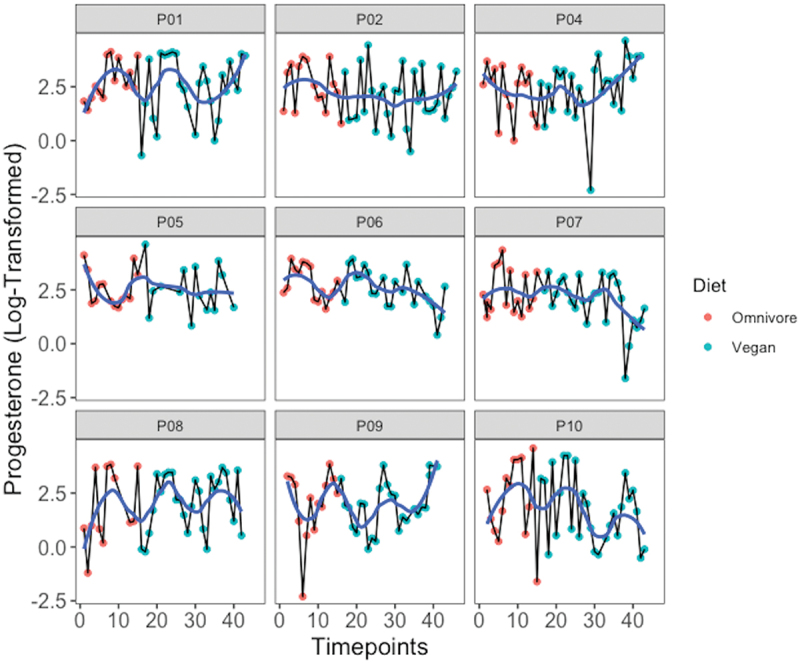
Progesterone values transformed to their natural logarithm over the 12 weeks for each individual. The blue line describes the locally weighted mean values (loess) to visualize time course.

The average cycle length was 30.29 ± 3.84 days with a bleeding phase of 5.24 ± 0.74 days. No significant changes in rhythm could be identified for E2 and P over time or treatment (Figures 3 and 4). One participant (P9) reported the cessation of the bleeding phase at the end of the last menstrual cycle after examination. However, no difference was found in hormone concentration between the omnivore and vegan phases in this individual.

### 3.5. Questionnaire

No significant differences were found between the omnivorous and vegan phases when analyzing the questionnaires.

## 4. Discussion

The aim of this study was to investigate the influence of a change to a vegan diet on body composition, physical performance, and menstrual cycle in recreationally trained women. A change to a vegan diet without specific requirements for macronutrient distribution leads to a significant decrease in protein intake (absolute and relative values) and a significant increase in carbohydrate intake (absolute and relative). In addition, a significant decrease in BW, SMM and CMJ was observed.

Current recommendations for fitness-oriented and individuals recreationally trained individuals in terms of macronutrient contribution are 45–55% carbohydrates (3–5 g/kg BW/day), 15–20% protein (0.8–1.2 g/kg BW/day), and 25–35% fat (0.5–1.5 g/kg/BW/day) [11,12,14]. Concerning the percentage change in energy intake of our individuals, there was a 6.1% decrease in total energy intake. In addition, the protein and fat intake decreased over time (protein: 15.5% to 13.6%; fat 39.5% to 32.8%), and the carbohydrate content increased from 45% to 53.6%. The percentage values for carbohydrates in both phases are within the general recommendations for healthy fitness-oriented individuals [11]. For fat intake, the percentage was above the recommendations during the omnivorous phase and only within the recommended range during the vegan phase [7,20].

However, general nutritional recommendations in sports are mainly given in g/kg bw for fitness-oriented individuals and athletes. The change in macronutrient distribution can also be seen in the relative values (g/kg bw) (Table 1) and is consistent with previous observations [20]. Compared to the percentage values, however, all relative values of the macronutrients in the omnivorous and vegan phases are between the recommendations of the ISSN [11].

Interestingly, a significant decrease in BW and SMM was observed. As a result, it appears that the relative protein intake during the vegan phase was not sufficient to maintain SMM. Presumably, the percentage intake also plays a role here. As described above, this was below the current recommendations for fitness-oriented individuals. Another aspect could be the insufficient intake of different plant protein sources, which led to a slight reduction in SMM. It is known that the majority of individual plant foods have a low biological value and lower amount of essential amino acids [36]. Exceptions are soy and quinoa, which can be easily combined to obtain a higher biological value [36]. Based on this, it can be speculated that a non-optimal amino acid profile and lower bioavailability led to a reduction in SMM.

However, it cannot be assumed that low muscle mass is present due to a plant-based diet. Initial studies show that with a balanced protein intake, similar muscle adaptations can be expected as with animal protein sources [15,16]. Probably the limiting factor for a vegan dietary change is the feasibility of implementing the recommendation in everyday life [37]. Although the participants were supervised by a nutritionist throughout the period and instructed to maintain the best possible energy intake and macronutrient distribution, the participants were unable to do this independently. It appears that closer supervision by a dietitian is necessary to achieve the appropriate amount and distribution in the amino acid profile to avoid negative effects on the SMM.

In terms of performance, a moderately significant treatment effect was found for the CMJ. Previous studies show no difference in performance between omnivorous and vegan athletes [38,39], as well as with dietary change in physically active individuals [20]. However, it is unlikely that the reduction in performance in the CMJ is related to the dietary change. In contrast to the omnivorous phase, the carbohydrate intake was higher during the vegan phase. In addition, no significant reduction in energy intake was observed, suggesting that an energy deficit can be ruled out. Moreover, during explosive movements such as the CMJ, only the phosphate stores are used as a primary energy source. As a result, no precise explanation can currently be given for the decline in the CMJ.

If the individual menstrual cycles of the participants are considered, all participants possessed an individual rhythm before the beginning of the study as well as during both dietary phases (Figures 3 and 4). There was no consistent phenomenon throughout the period. Only one participant reported the absence of the bleeding phase after the end of the intervention.

After reviewing the dietary records, no correlation could be found between the dietary change and the absence of the bleeding phase. It is important to note that all participants had adequate caloric and fat intake throughout the study. Extreme caloric deficits or insufficient fat intake may lead to possible disturbances in the formation of steroid hormones such as E2 or P [40]. It is well known that a relative energy deficiency syndrome (RED-S) can develop especially in female athletes [40]. This can lead to health as well as performance-related deficits [41]. At the same time, studies show that people on a vegan diet have a lower caloric intake than those on an omnivore diet [7]. Therefore, special care must be taken by professionals to ensure that recreationally trained individuals and athletes on a vegan diet do not increase the risk of developing RED-S. Although a change in macronutrient distribution can be observed in this study, no general influence on the menstrual cycle can be detected. However, it must be explicitly highlighted that the vegan phase was only carried out for eight weeks.

In summary, it is not enough for recreational athletes to be informed and aware of the recommendations for a sport appropriate diet in order to follow them on their own. It seems that the relative requirements for macronutrients, energy intake, and even food selections need to be regularly reviewed by an expert. Especially when changing dietary habits to a vegan diet, significant changes in macronutrient distribution could be observed, which might influence SMM. However, with sufficient protein intake, similar adaptations can be achieved with a vegan diet as with an omnivorous diet.

## 5. Limitations

Although this study provides important and currently unique results, it is limited by the small sample size. Only 10 participants were recruited for this study. Therefore, the results cannot be interpreted as definitive findings. However, statistical power is not solely dependent on sample size, but also on the number time points measured. There were a total of 84 measurement time points for nutrition and more than 40 for hormones. Based on this, a time series analysis could be performed, which is also used in doping analysis to determine individual processes or phenomena. In addition, the food was not analyzed for quality during the two phases. Both animal and pure plant products may have undergone a high number of processing steps, which lowers the quality of the food. Thus, a change could probably have occurred here as well due to the change in diet. However, due to capacity constraints, a sub-analysis was not possible and should be included in future investigations. Furthermore, the study design used also has some limitations. Only the change from omnivore to vegan was examined. The effects of a change from vegan to omnivore cannot be answered. Further studies should use a cross-over study design to investigate these effects. In addition, it would be interesting to examine whether the change in macronutrient distribution and SMM could be reversed by a further switch from vegan to omnivore. As a final aspect, no conclusions can be drawn about competitive athletes due to their level of performance and training frequency.

## 6. Conclusion

The purpose of this study was to evaluate the effects of a vegan dietary transition on body composition, performance, and menstrual cycle in recreationally trained women. It was observed that the dietary transition resulted in changes in protein and carbohydrate intake, which is consistent with previous studies conducted in physically active individuals and the general population. It was also found that this resulted in a slight decrease in SMM. The dietary change had no clear effect on performance and menstrual cycle. It is important to note that only eight weeks were observed and no conclusions can be drawn regarding long-term effects at this time. Future research on vegan diets, particularly in female athletes, is necessary to provide more accurate assessments.

## Data Availability

Anonymized raw data can be viewed and obtained upon request to the corresponding author.
